# Impact of the HOP-UP-PT program on older adults at risk to fall: a randomized controlled trial

**DOI:** 10.1186/s12877-021-02450-0

**Published:** 2021-10-01

**Authors:** Sara K. Arena, Christopher M. Wilson, Lori Boright, Edward Peterson

**Affiliations:** 1 Physical Therapy Program, Oakland University, School of Health Sciences, Human Health Bldg, 433 Meadowbrook Road, Rochester, MI 48309-4401 USA; 2 Henry Ford Health System, Department of Public Health Sciences, Detroit, MI USA

**Keywords:** Home-based, Older adult, Prevention, Upstreaming, Physical therapy, Falling, Independent living

## Abstract

**Background:**

Reduced falls and fall risks have been observed among older adults referred to the HOP-UP-PT (Home-based Older Persons Upstreaming Prevention-Physical Therapy) program. The purpose of this study was to describe outcomes of HOP-UP-PT program participants and then to compare these outcomes to non-participants.

**Methods:**

Six Michigan senior centers referred adults ≥65 years who were at-risk for functional decline or falls. 144 participants (*n* = 72 per group) were randomized to either the experimental group (EG) or the control group (CG). Physical therapists (PTs) delivered physical, environmental, and health interventions to the EG over nine encounters (six in-person, three telerehabilitation) spanning seven months. The CG participants were told to continue their usual physical activity routines during the same time frame. Baseline and re-assessments were conducted at 0-, 3-, and 7-months in both groups. Descriptions and comparisons from each assessment encounter were analyzed.

**Results:**

Participants ages were: EG = 76.6 (7.0) years and CG = 77.2 (8.2). Baseline measures were not significantly different apart from the Short Physical Performance Battery (SPPB) which favored the EG (*P* = 0.02). While no significant differences were identified in the survey outcomes or home environment assessments, significant differences in favor of the EG were identified in common fall risk indicators including the Timed Up and Go (*P* = 0.04), Four Test Balance Scale (*P* = 0.01), and the modified SPPB (*P* = 0.02) at the 3-month assessment visit. However, these differences were not sustained at the 7-month assessment as, notably, both groups demonstrated positive improvements in the Four Test Balance Score and SPPB. For individuals at a moderate/high fall risk at baseline, 47.8% of CG reported falling at seven months; whereas, only 6.3% of EG participants meeting the same criteria reported a fall after HOP-UP-PT participation.

**Conclusions:**

A prevention-focused multimodal program provided by PTs in older adults’ homes proved beneficial and those with the highest fall risk demonstrated a significant decrease in falls. A collaboration between PTs and community senior centers resulted in upstreaming care delivery that may reduce both the financial and personal burdens associated with falls in an older adult population.

**Trial registration:**

This study was retrospective registered at Clinical Trials.gov, TRN: NCT04814459 on 24/03/2021.

**Supplementary Information:**

The online version contains supplementary material available at 10.1186/s12877-021-02450-0.

## Background

Older adults generally have a desire to continue living in their own home, a term commonly referred to as aging-in-place (AIP) [1]. Programs focused on AIP for frail older adults have demonstrated an annual reduction in number of days in the hospital by 46% and nursing home costs by 54% [2, 3]. There is also evidence for improved functional status, medication compliance, disease knowledge, and overall satisfaction when implementing AIP interventions [4, 5]. Programs aimed at addressing the AIP-needs of older adults should include simple identification criteria, provide long-term intervention strategies, utilize tools capable of evaluating the most relevant concerns, employ technology to empower older adults, and evaluate cost-effectiveness of the intervention [6].

Falls present a barrier to safe AIP. In the US, falls and expenses associated with emergency room visits, hospitalizations, and fatalities have disproportionately affected those over the age of 65 [7, 8]. Annually, one in three older adults report a fall and medical attention is required for nearly a third of these individuals; yet less than half report these occurrences to their physician [7–10]. Furthermore, once an individual falls, they are likely to do so again [11]. Given an anticipated population growth of older adults over the next decade, there is an urgent need to implement upstream approaches to reduce current fall rates [8, 12].

Older adult programming that primarily delivers exercise, including *Matter of Balance* or the *Otago Exercise Program* (OEP), have evidence of their effectiveness in reducing fall rates but do not comprehensively address other key factors [13–16]. An Australian based program, *Stay on your Feet,* has demonstrated cost savings when using a multimodal approach that includes physical activity, balance interventions, home modifications, medication review, and use of appropriate eyewear [17]. Additionally, the *Community Aging in Place, Advancing Better Living for Elders (CAPABLE)* program uses a multimodal, interdisciplinary approach including occupational therapists, registered nurses, and handymen to bring about significant cost savings; however, CAPABLE does not include individualized balance exercises [18, 19]. While each program has elements of successful strategies, further examination of novel partnerships and referral patterns that leverage public health and medical practice models may prove to add evidence for the systematic change necessary to facilitate fall prevention and AIP. Furthermore, a critical review of existing literature did not reveal consistent use of common assessment tools or outcome measures in determining effectiveness of fall prevention programming, though multimodal approaches delivered by health care professionals was determined to be a consistent theme.

The Home-based Older Persons Upstreaming Prevention Physical Therapy (HOP-UP-PT) program also uses a multimodal approach; however, a unique component of this program is that referrals to the program are not from medical professionals, but from local senior community centers (SCC). To the author’s knowledge, the HOP-UP-PT program is the first program to utilize this referral approach. Three prior publications; a pilot, observational, and pilot long-term impact study [20–22] provide emerging evidence that this referral mechanism, followed by subsequent physical therapist (PT)-led functional, health, and environmental assessments and interventions can reduce falls and fall risk [20–22]. Specifically, significant improvements in measures of functionally associated fall risk (Timed Up and Go, Four Stage Balance Test, and STEADI Fall Risk Level), environmental risk (HOME FAST), and fall self-efficacy and health behavior surveys were identified in 30 older adults after participating in the HOP-UP-PT program [21]. Due to the long-term relationships built between SCC staff members and their older adults patrons, SCCs are well positioned to identify older adults at risk of a functional decline [21] and to then refer individuals directly to a trained HOP-UP-PT provider as was evident in these prior publications [20–22]. Furthermore, SCC staff may have insights into the social determinants that increase the risk of falls or inability to AIP and can provide resources (e.g., transportation, Meals on Wheels) to address these needs. This referral mechanism leverages the ability of a licensed PT to serve as a healthcare access point from which communication to physicians or other healthcare providers can be initiated [20]. Additionally, during and after the 7-month HOP-UP-PT program, participants and PTs collaborate with the local SCC to identify and initiate beneficial services (e.g., exercise classes, home repair services) for the older adult.

While there has been prior observational evidence supporting the positive benefits of HOP-UP-PT, a randomized controlled trial (RCT) examining the impact of this program is warranted. This study will examine the specific effects of the program’s multimodal features and referral processes with an older adult population at-risk for falls or difficulty with AIP. The purpose of this study was to describe outcomes of program participation and then to compare outcomes of HOP-UP-PT program participants to non-participants. We hypothesize older adult HOP-UP-PT participants will have reduced falls and fall risks compared to their non-participant counterparts.

## Methods

### Research design

After securing Institutional Review Board (IRB) approval from Oakland University (#912215), a RCT included older adults from six SCCs throughout Southern Michigan USA (*n* = 24 from each location). The study was retrospective registered at Clinical Trials.gov, TRN: NCT04814459 on 24/03/2021. Specific details of the trial design are included as a supplement file to this manuscript. No changes were made to the approved IRB study methods or the clinical trial during data collection.

### Participants

The six SCCs (Auburn Hills, Novi, Saline, St. Clair Shores, Pittsfield, and Van Buren County) are supported by their local public municipalities to provide services to older adults within their respective service regions. The SCC staff were asked to use professional judgement and the *Stay Independent Brochure Questionnaire-Risk of Falling* to identify and refer older adults at risk for falls or difficulty with AIP [23]. SCC staff included department directors, program coordinators, and/or center staff who had been trained in the *Stay Independent Brochure Questionnaire* and were delegated this responsibility by the SCC director. While objective referral criteria were not mandated for use by SCC staff when determining potential program candidates, prior evidence found that SCC staff were indeed able to identify individuals “at risk” for future functional decline using the suggested questionnaire to guide the decision to refer [21]. However, the authors recognize selection variation and decisional bias by SCC staff is possible and imposes a potential selection limitation.

Study participants were included if they were [1] greater than or equal to 65 years of age, [2] SCC staff identified them as ‘at-risk’ for decline in community dwelling status due to physical, social, economic, or community-related barriers, and [3] willing to participate in the HOP-UP-PT program. Participants were excluded if they [1] received physical therapy services within the prior two months in any setting, [2] had been hospitalized within the prior two months, or [3] were currently receiving palliative or hospice care. These exclusion criteria were chosen to assure the individual was not actively experiencing an acute or subacute healing condition that would best align with rehabilitative care models and therefore, would be best positioned to receive the benefit of prevention-focused services. Additionally, participants were excluded if the initial evaluation by the licensed PT suggested that the person’s cognitive status (as determined by the Mini Cog [24] or Trail Making Test Part B [25]) or medical status (as determined by the American College of Sports Medicine [ACSM] exercise preparticipation health screening [26]) would not permit safe PT examination or interventions without further physician assessment.

### Sample size justification

Results of a prior observational study [21] using a matched population identified a mean fall risk classification in the Stop Elderly Accidents, Deaths, and Injuries (STEADI) [27] program of 0.9 +/− 0.7. Therefore, a decrease in the STEADI risk category to 0.35 would have more than 90% power, at an adjusted two-sided 0.017 alpha value, to detect differences in a sample of 100 total participants allocating 50 individuals to each group. Given the potential for participant attrition and analysis of other variables, enrollment of 144 participants was planned.

### Procedures and measures

Each SCC was allotted 24 enrollment spots; 12 were assigned to an experimental group (EG) who participated in the 7-month HOP-UP-PT program and 12 were assigned to the control group (CG) that included only baseline, 3-month, and 7-month assessments. The CG received 3 in-person assessment visits (baseline, 3-month, 7 month), but were instructed to continue their normal level of activity throughout the 7-months after which they were offered the opportunity to receive the HOP-UP-PT program. This study reports only the 7-month time frame during which the EG and CG can be directly compared.

Participant recruitment was conducted between March and September 2019 at which point the intended study size had been enrolled. The results reported in this study were collected through June of 2020, with results of the larger study methods completed by March of 2021. Within each SCC, the first participant was randomized to either the EG or CG and subsequent participants from that SCC were distributed in an alternating order of referral receipt and blinded to the SCC staff during the group assignment process (i.e., participant #1 assigned to EG, participant #2 CG, participant #3 EG). Exceptions to this process were made when participants lived in the same home (e.g., spouses, siblings, housemates, parent-child) to reduce participant observation bias. In these situations, both participants were assigned to the same group using the next open spot (i.e., if the next spot was a CG, both were assigned to this group and the next referral was back-filled to the EG.) Once participants were assigned to either the EG or CG, it was no longer feasible to blind data collectors, participants, or SCC staff to groupings.

**Fig. 1:**
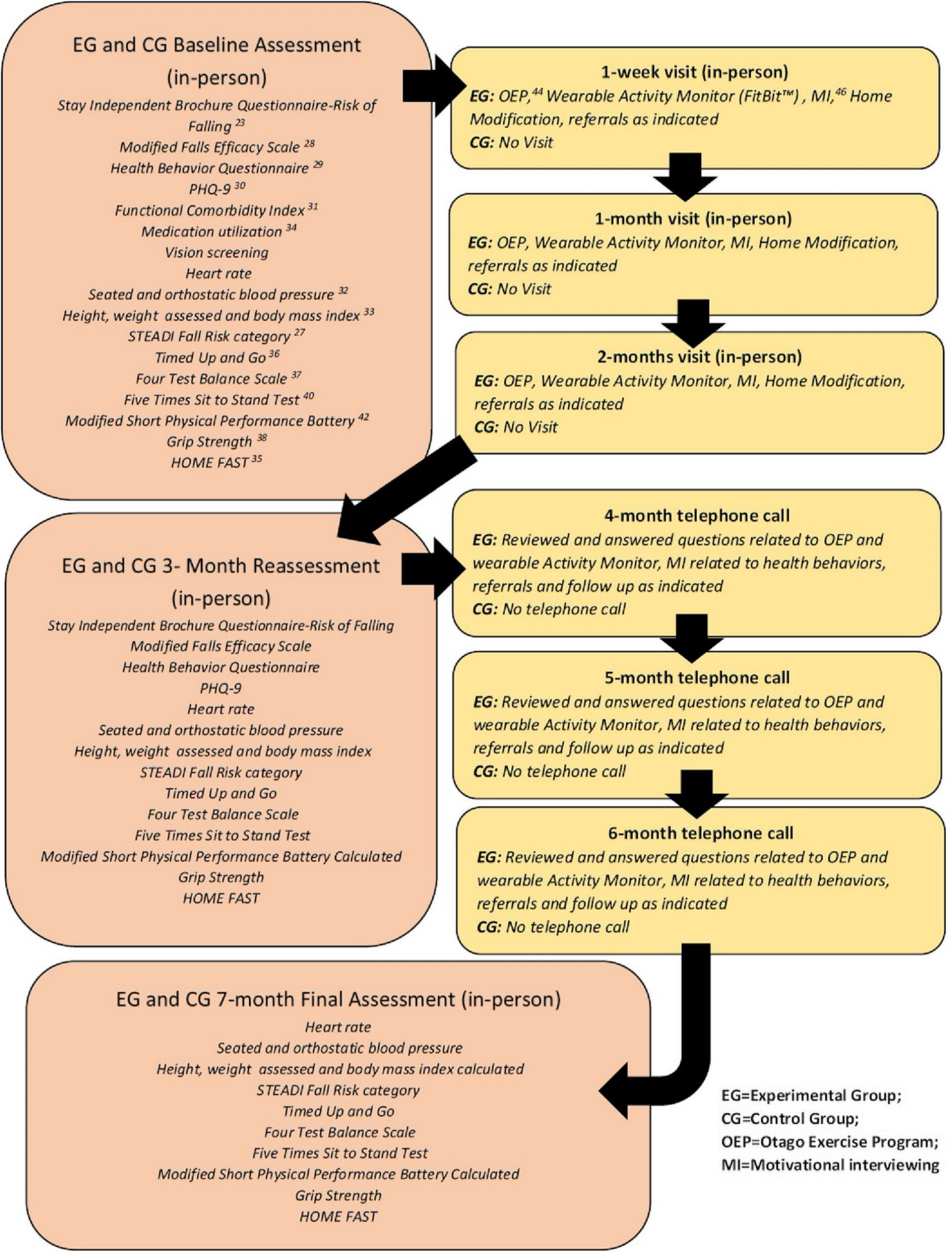
Key assessments and interventions performed during each encounter

The study protocol has been previously described by Wilson et al. [20] and Arena et al. [21] and involves assessment and intervention via a total of six in-person home visits and three telerehabilitation phone visits by a licensed PT during a 7-month time frame. The details of the EG and CG timeline and the associated program assessment tools and interventions are detailed in Fig. 1.

### Key outcome metrics

#### Surveys

The *Stay Independent Questionnaire*, [23] the *Modified Falls Efficacy Scale*, [28] and a health behavior questionnaire were administered to garner insight of participants’ current behaviors related to physical activity, consuming fruits and vegetables, not smoking, and being at a recommended weight [29]. Additionally, the *Patient Health Questionnaire (PHQ-9)* quick depression screen was administered to assess the mental and emotional health of participants [30]. The *Stay Independent Questionnaire* was selected as it is a component of the STEADI Algorithm [27] so was already an included aspect of fall risk assessment and stratification of each participant. The *Modified Falls Efficacy Scale* and *PHQ-9* were selected for their favorable metrics and each measure’s ability to be administered in a concise time frame. Additionally, while moving the bar on fall frequency counts may be difficult, these measures may offer insight into other domains of fall risk that may prove beneficial. As a limited number of participants completed these forms upon program completion, only the baseline and 3-month encounters are described in this manuscript.

#### Health metrics

The Functional Comorbidity Index, [31] current medication use, heart rate, height, and weight were assessed at each encounter as well as seated, supine, and standing blood pressure (BP) [32]. A baseline body mass index (BMI) was calculated using the Center for Disease Control and Prevention’s Adult BMI Calculator [33].

#### Short Physical Performance Battery calculation

The Short Physical Performance Battery (SPPB) is an assessment of the lower extremity function of an older person [42]. In a systematic review, SPPB baseline score of 4 to 6 has an increased risk of developing a future disability by 2.9 to 4.9 times and that lower SPPB scores increased the risk of institutionalization and functional decline [43]. In addition, it has predictive value for hospitalization and death when an individual has lower scores (0–4) compared to higher scores [8–12] [44]. The SPPB is calculated using the collective outcomes of the Four Stage Balance Test, [37], the 3-m gait speed [45], and the 5XSTS, [41] in that order. Each of the three tests has a highest possible score of 4 for a best possible score of 12 and a lowest possible score of 0. The OEP utilizes the Four Test Balance Scale (not the Four Stage Balance Test) to prescribe exercise categories with the main difference being that the Four Test Balance Scale does not use the “semi-tandem stance 3-9.99 seconds hold” category. As the OEP was a key intervention in the HOP-UP-PT program, a modified SPPB was calculated. Specifically, participants who held a semi-tandem position for 10 s but could not hold a tandem stance for 10 s only achieved a total score for that SPPB category of 2. The authors recognize this may result in a lower-than-actual SPPB score for all group means. Therefore, the scoring was as follows: Side-by-side stand (e.g., feet together) held for 10 s = 1-point, semi-tandem stand held for 10 s = 1-point, and tandem stand held for 10 s = 2-points.

For the 3-m gait speed assessment, the test was conducted twice and the fastest time in seconds was used for scoring. Scoring was as follows: unable to complete = 0-points, more than 6.52 s = 1-point, 4.66 to 6.52 s = 2-points, 3.62 to 4.65 s = 3-points, less than 3.62 s = 4-points. This resulted in 4 points being the maximum score for this category. For the 5XSTS test, it was conducted once and was scored using seconds as the unit of measure. The participant was to complete the test without using their arms to assist with standing. Scoring was as follows: unable to complete or took longer than 60 s = 0-points, 16.70 s to 60 s = 1-point, 13.70 to 16.69 s = 2-points, 11.20 to 13.69 s = 3-points, 11.19 s or less = 4-points.

#### STEADI fall risk categorization

The 2017 version of the STEADI Algorithm was used to categorize participants as low, moderate (mod), or high fall risk [27]. The STEADI Algorithm uses a combination of a screening questionnaire, [23] review of medical history and medications, a home assessment, functional assessments, and fall frequency to stratify risk of future falls. While the STEADI Algorithm underwent revisions since the study onset, the 2017 version was utilized as a guide for key outcome metrics reported in this study. Clinical measures collected included: categorization as low, mod, or high fall risk (via STEADI), orthostatic hypotension BP measures, [32] corrective eyewear use, medication consumption, [34] assessment of environmental safety (via HOME FAST), [35] Timed Up and Go (TUG), [36] Four Test Balance Scale (from the OEP protocol), [37] grip strength (via handgrip dynamometry), [38] and the 5XSTS [39–41]. The fall risk category was determined and recorded by the PTs at each of the three assessment encounters. The timing of measurement and delivery for each of these stated measures is detailed in Fig. 1.

#### HOME FAST 

The HOME FAST tool was used to assess participant home safety and modification needs [35]. The tool evaluates 25 home safety domains and includes questions of lighting, floor surfaces, and properly fitted footwear. Twelve of the home safety questions are scored as either Yes (indicating the recommended safety modifications were present) or No (indicating the recommended safety modifications had not been met). An additional 13 questions have an N/A option to be used in circumstances where a condition was not met (e.g., participant does not have a pet or stairs in the home). The investigators coded the responses as YES = 1-point, NO = 2-points, and N/A = null with 0 points assigned. Therefore, overall HOME FAST scores that decrease over time would suggest diminished home fall and accident risks.

### Key interventions

Interventions provided to EG participants during the visits included [1] the OEP [46, 47] which is a well-established exercise program with evidence that it reduces falls among community-dwelling older adults, [2] motivational interviewing (MI) to optimize positive health behaviors, [48] and [3] home and environmental modification recommendations aimed at safety. Participants were provided with and educated on the use of a wrist-worn activity tracker (Fitbit Alta; Fitbit, Inc., San Francisco, CA) and an automated BP monitor unit (Omron HEM-712C Automatic Inflation Blood Pressure Monitor; Omron Corporation, Kyoto, Japan). Participants were educated in the use and benefit of both the activity tracker and BP monitor for self- monitoring; however, compliance nor the associated outcome data were collected. Finally, when follow up needs were identified (e.g., orthostatic hypotension, community exercise classes), these referrals were made and documented. Figure 1 describes placement of these key interventions for EG participants.

### Data analysis

Descriptive statistics were generated to analyze demographic and outcome variables of both the EG and CG. The baseline encounters for the EG (E1) and CG (C1) were compared to 3-month encounters (E2 and C2) and 7-month encounters (E3 and C3) via a two-sample Wilcoxon test (for continuous or ordinal variables) and a Chi-squared test (for binary variables). The Wilcoxon test was preferred as the majority of the variables were not normally distributed. We tested normality using the Shapiro-Wilk test. As adjusted means were utilized for interval changes, 95% confidence intervals were included. Statistical analysis was performed using SAS v.9.4 (SAS Institute, Cary, NC) software for Windows with significance determined at *P* < .05.

## Results

**Fig. 2:**
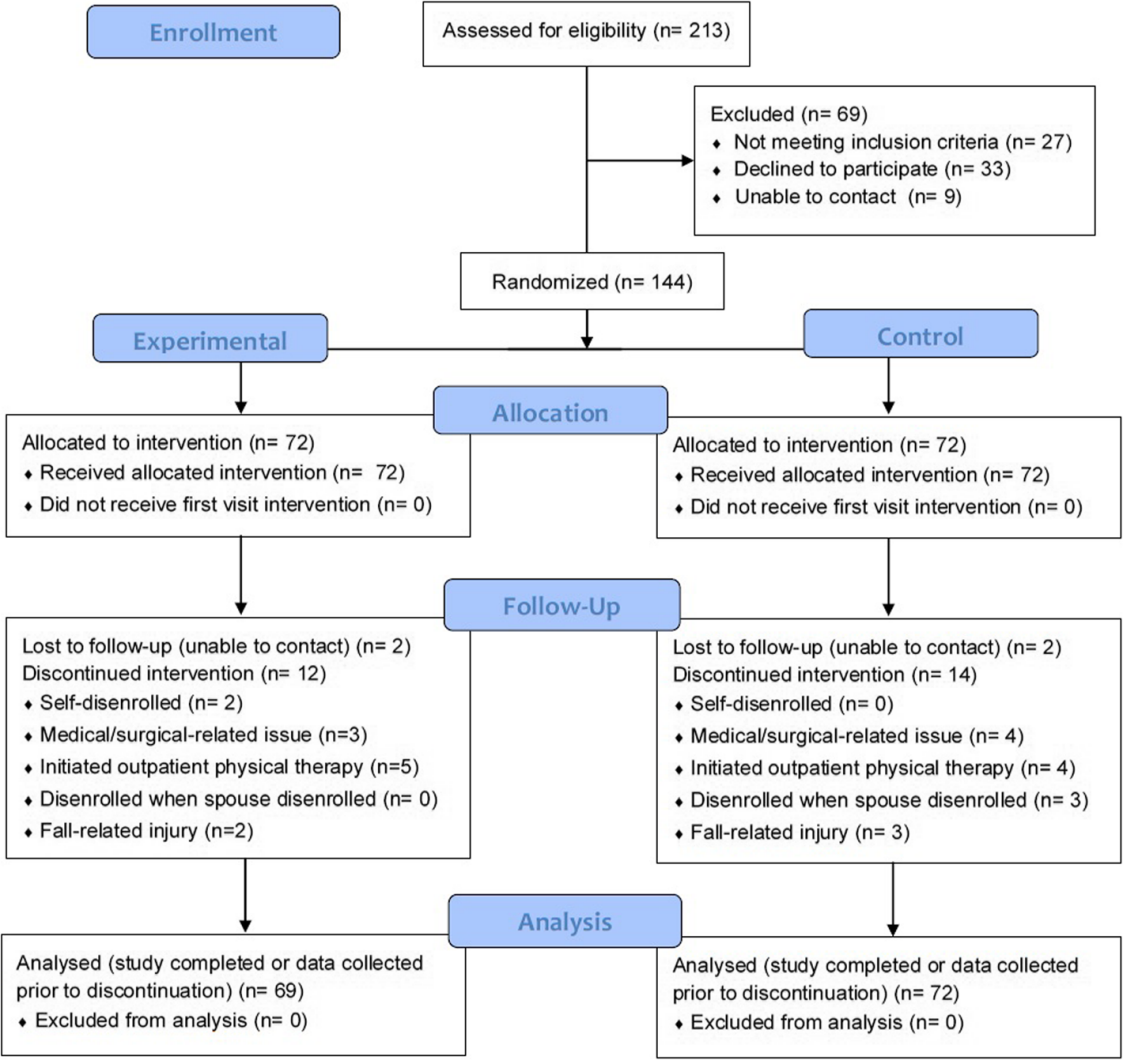
CONSORT Flow Diagram

SCC staff referred 213 individuals to the HOP-UP-PT program. See the CONSORT Flow Diagram (Fig. 2) for the number of individuals screened, enrolled, and analyzed. Ultimately, 69 were not enrolled resulting in enrollment of 144 total participants (EG = 72 and CG = 72), 24 from each of the six SCCs. Participant baseline demographics are reported in Table 1.

**Table 1:** Baseline experimental and control group demographics

	Experimental Group	Control Group	
	n=	n=	
Females	57	53	
Males	15	19	
Medication Consumption			
*≤ 4*	15	27	
*Between 5 and 9*	35	29	
*> 9*	19	15	
*psychotropic drug category*	18	10	
Use of corrective lenses	58	60	
	Mean (SD)	Mean (SD)	*P*-value
Age	76.6 (7.0)	77.2 (8.2)	0.51
Functional Co-morbidity Index	4.0 (2.1)	3.9 (2.3)	0.64
Weight (lbs.)	174.8 (33.5)	168.8 (37.9)	0.24
Height (inches)	64.0 (3.6)	64.0 (4.0)	0.99
Body Mass Index	29.9 (5.4)	28.6 (5.4)	0.12
Systolic Blood Pressure (mmHg)	133 (20)	131 (15)	0.52
Diastolic Blood Pressure (mmHg)	76 (10)	78 (10)	0.90
Heart Rate (beats/minute)	70 (10)	71 (10)	0.78
STEADI risk category (0 = low,1 = moderate, 2 = high)	0.77 (0.7)	0.71 (0.7)	0.59

*SD = Standard deviation*

No participants were disenrolled or reported harms or unintended effects as an outcome of study participation. Fourteen EG and 16 CG participants were disenrolled during the study. See Fig. 2 for reasons for disenrollment. It is noteworthy that of the two EG participant requiring disenrollment for a fall-related injury, one was categorized as a high risk and had the fall occur during the first month of participation (before appreciable interventions could be administered) and the second participant was categorized as low risk and fell while vacationing.

### Reported falls

The percentages of participants reporting at least one fall in the past year at baseline was EG = 51.4% and CG = 47.2% (*P* = 0.62). Reported falls since the prior assessment at 3-months was EG = 7.8% and CG = 5.2% (*P* = 0.72), while at 7-months, EG = 13.0% and CG = 26.8% (*P* = 0.07). While a slightly higher percentage of individuals reporting one fall at the 3-month encounter was observed in the EG (compared to CG), the total number of fall occurrences was significantly lower in the EG compared to the CG (*P* = 0.049). In other words, CG participants who fell did so more frequently.

A secondary analysis of falls among participants classified as mod and high risk (via STEADI) found no difference at the baseline (*P* = 0.58) or the 3-month encounter (*P* = 0.24). However, at the 7-month encounter, a significant reduction in falls (*P* = 0.01) was reported by EG participants (6.3%) compared to CG participants (47.8%), This finding suggests an 8-fold reduction in falls for mod and high-risk HOP-UP-PT participants compared to older adults who did not participate in the program.

### Outcomes of HOP-UP-PT program participants

Table 2 provides detail of the change in the outcome variables for the EG participants between the E1 and E2 and E2 and E3 encounters and the CG participants between the C1 and C2 and C2 and C3 encounters. The number of participants (n) varies in baseline data measures in both the EG and CG for the 72 enrolled participants as some data was not recorded by the data collector at this encounter. While the rationale for data omission is unknown human error, participant declining test, and space, equipment or environmental factors are likely contributors in field testing. Furthermore, at the 3- and 7-month encounters participant counts are impacted by disenrollment and missed visits.

**Table 2:** Description of change in experimental (E) and control (C) groups between the three assessment encounters

Variable	N	E1-E2 encounter change Mean (SD)	P-Value	N	E2-E3 encounter change Mean (SD)	P- Value	N	C1-C2 encounter change Mean (SD)	P-Value	N	C2-C3 encounter change Mean (SD)	P- Value
Systolic Blood Pressure	64	−5.4 (18.2)	0.05	54	−4.4 (19.9)	0.06	59	−6.2 (80.0)	***0.01***	51	−2.9 (14.1)	0.12
Diastolic Blood Pressure	64	−0.6 (15.9)	0.11	54	−1.6 (9.4)	0.13	59	−1.9 (12.8)	0.07	51	−3.1 (11.7)	0.06
Heart Rate	64	1.1 (8.6)	0.36	53	3.1 (8.5)	***0.01***	56	2.8 (10.7)	***0.05***	49	0.6 (9.3)	0.38
Grip Strength	60	−0.5 (6.8)	*0.69*	54	0.5 (6.3)	0.46	58	−0.9 (7.9)	0.48	47	0.3 (2.7)	0.65
Timed Up and Go	64	−0.6 (4.1)	***0.001***	53	−0.7 (3.5)	***0.04***	58	−0.1 (2.6)	0.17	50	−0.9 (3.7)	0.06
Gait Speed	64	−0.2 (1.3)	***0.001***	53	−0.5 (1.2)	***0.001***	58	−0.1 (1.2)	***0.04***	50	−0.5 (2.3)	0.06
Four Stage Balance Test	65	0.4 (0.8)	***0.001***	50	0.3 (0.9)	***0.02***	56	−0.1 (0.7)	0.50	46	0.1 (0.9)	0.39
Five Time Sit to Stand	61	−2.2 (3.6)	***0.001***	48	−3.1 (3.9)	***0.001***	47	−0.5 (5.6)	***0.03***	41	−0.7 (4.8)	0.06
SPPB	64	0.9 (2.3)	***0.001***	47	1.5 (2.3)	***0.001***	53	0.8 (1.9)	***0.01***	45	1.1 (2.3)	***0.001***
STEADI Fall Risk	61	−0.2 (0.7)	***0.03***	49	−0.4 (0.7)	***0.001***	55	−01 (0.4)	0.51	46	−0.1 (0.6)	0.31
HOME FAST	53	−0.3 (1.52)	0.1	46	−0.5 (2.4)	0.16	45	0.04 (3.7)	0.25	40	−0.2 (6.2)	0.98

*E1 = experimental group baseline assessment; E2 = experimental group 3-month assessment; E3 = experimental group 7-month assessment; C1 = control group baseline assessment; C2 = control group 3-month assessment; C3 = control group 7-month assessment; SD = standard deviation; SPPB = Short Physical Performance Battery. Items reaching statistical significance on P < 0.05 are italic and bold*

### Comparison of survey outcome measures between groups

The results of the baseline (E1/C1) and 3-month (E2/C2) *Stay Independent Questionnaire*, *Modified Falls Efficacy Scale*, and *Health Behavior Questionnaire,* and *PHQ-9* outcomes are reported in Table 3 but were not significantly different between groups.

**Table 3:** Comparison of surveys outcomes between groups at baseline and 3-month assessments

		Encounter	n=	Mean (SD)	Encounter	n=	Mean (SD)	P-Value
		Control Group			Experimental Group			
Stay Independent Brochure Questionnaire-Risk of Falling • 0-Lowest Perceived Risk • 14-Highest Perceived Risk		C1	69	4.7 (3.2)	E1	72	4.8 (3.0)	0.84
		C2	48	4.2 (2.8)	E2	58	3.3 (2.9)	0.10
Modified Falls Efficacy Scale • 0-Lowest Confidence • 14-Highest Confidence		C1	68	9.0 (1.8)	E1	72	9.2 (1.7)	0.82
		C2	47	9.2 (1.7)	E2	57	9.7 (1.1)	0.39
Health Behavior Questionnaire *Transtheoretical Model-Stages Behavior of Change* Precontemplation = 5 Contemplation = 4 Preparation = 3 Action = 2 Maintenance = 1	Meets Recommended Physical Activity Levels	C1	69	1.9 (1.3)	E1	70	2.0 (1.2)	0.25
		C2	47	2.2 (1.5)	E2	53	1.7 (1.0)	0.23
	Consumes Recommended Fruits and Vegetables	C1	66	2.4 (1.5)	E1	71	2.2 (1.3)	0.52
		C2	47	2.2 (1.5)	E2	52	2.0 (1.2)	0.59
	Abstains from Smoking	C1	68	1.1 (0.4)	E1	72	1.1 (0.5)	0.54
		C2	47	1.1 (0.4)	E2	53	1.0 (0.3)	0.92
	At Recommended Weight	C1	68	2.1 (1.4)	E1	67	2.2 (1.3)	0.62
		C2	46	1.9 (1.2)	E2	52	1.8 (1.2)	0.79
Patient Health Questionnaire-9 (PHQ-9) • 0- Lowest Severity of Depression • 27-Highest Severity of Depression		C1	69	4.0 (4.3)	E1	70	3.4 (3.9)	0.38
		C2	47	2.6 (3.2)	E2	57	2.6 (3.1)	0.77

*C1 = control group baseline assessment; C2 = control group 3-month; E1 = experimental group baseline assessment; E2 = experimental group 3-month assessment; SD = standard deviation*

### Comparison of health assessments between groups

No significant difference was identified in the *Functional Co-morbidity Index*, heart rate, systolic or diastolic seated BP, orthostatic hypotension, weight, height, or BMI between the three assessment encounters.

### Comparison of fall risk and functional assessment outcomes between groups

Comparisons of key fall risk and functional outcomes from EG and CG participants as well as those with elevated fall risk (via STEADI) are detailed in Table 4.

**Table 4:** Comparison of baseline, 3-, and 7-month assessment fall risk and functional outcomes

Outcome Measure		TUG Seconds to complete test		Four Test Balance Scale 1 = Feet together 10 s 2 = Semi-tandem 10 s 3 = Tandem 10 s 4 = One leg Stance 10 s		Chair-Stand Test Seconds to perform 5 reps		Gait Speed Seconds to walk 3 m		*Short Physical Performance Battery 0 = lowest score 12 = highest score		Grip Strength Measured in Kg	
		Mean(SD)	P-Value	Mean(SD)	P-Value	Mean(SD)	P-Value	Mean(SD)	P-Value	Mean(SD)	P-Value	Mean(SD)	P-Value
All Participants	E1	11.5 (4.6) *n* = 71	0.09	2.7 (0.8) *n* = 72	0.31	15.5 (4.8) *n* = 68	0.50	4.1 (1.8) *n* = 71	0.21	8.4 (2.7) *n* = 71	***0.02***	21.9 (11.1) *n* = 72	0.55
	C1	13.3 (6.0) *n* = 70		2.4 (1.0) *n* = 70		16.4 (6.5) *n* = 61		4.8 (3.1) *n* = 70		7.3 (2.9) *n* = 71		22.3 (9.7) *n* = 69	
	E2	10.7 (3.7) *n* = 65	***0.04***	3.0 (0.8) *n* = 65	***0.01***	13.1 (3.9) *n* = 62	0.06	3.8 (1.7) *n* = 65	0.17	9.3 (2.5) *n* = 65	***0.02***	22.1 (9.3) *n* = 60	0.93
	C2	13.0 (6.3) *n* = 57		2.5 (0.9) *n* = 55		15.5 (6.4) *n* = 50		4.5 (2.6) *n* = 57		8.0 (3.0) *n* = 53		21.6 (8.1) *n* = 58	
	E3	10.7 (3.7) *n* = 54	0.35	2.9 (2.8) *n* = 50	0.14	12.7 (3.5) *n* = 49	0.22	3.5 (1.4) *n* = 54	0.27	9.5 (2.2) *n* = 48	0.37	21.6 (10.0) *n* = 54	0.53
	C3	11.9 (5.4) *n* = 50		2.6 (0.9) *n* = 47		14.7 (6.5) *n* = 44		3.9 (1.8) *n* = 50		9.0 (2.6) *n* = 45		22.2 (8.6) *n* = 48	
Moderate and High Risk STEADI Category Participants	E1	12.9 (5.3) *n* = 42	0.07	2.4 (0.7) *n* = 43	0.17	16.7 (5.3) *n* = 39	0.53	4.7 (1.9) *n* = 42	0.16	7.6 (2.8) *n* = 42	***0.03***	19.3 (7.6) *n* = 43	0.39
	C1	15.7 (6.8) *n* = 38		2.0 (1.0) *n* = 39		18.2 (7.7) *n* = 30		5.9 (3.8) *n* = 38		6.2 (2.9) *n* = 39		20.6 (7.7) *n* = 38	
	E2	12.0 (4.3) *n* = 29	0.13	2.7 (0.7) *n* = 29	***0.01***	14.6 (4.1) *n* = 26	0.28	4.1 (1.6) *n* = 29	0.32	8.2 (2.4) *n* = 29	***0.03***	18.8 (8.0) *n* = 7	0.11
	C2	15.4 (7.5) *n* = 31		2.1 (0.8) *n* = 31		17.6 (8.0) *n* = 25		5.2 (3.1) *n* = 31		6.6 (3.0) *n* = 30		21.7 (8.2) *n* = 32	
	E3	13.8 (4.3) *n* = 15	0.99	2.5 (0.8) *n* = 14	0.06	14.7 (2.6) *n* = 12	0.66	3.9 (1.1) *n* = 15	0.49	7.6 (2.2) *n* = 14	0.61	19.1 (10.5) *n* = 16	0.17
	C3	14.6 (6.8) *n* = 22		2.0 (0.8) *n* = 20		18.0 (8.3) *n* = 19		4.5 (2.1) *n* = 22		7.3 (2.7) *n* = 20		21.2 (9.2) *n* = 22	

E1 = experimental group baseline assessment; E2 = experimental group 3-month assessment; E3 = experimental group 7-month assessment; C1 = control group baseline assessment, C2 = control group 3-month assessment, C3 = control group 7-month assessment; TUG = timed up and go test SD = standard deviation. Items reaching statistical significance on P < 0.05 are italic and bold. *Modified Four Test Balance Scale

### Environment assessment outcomes

While no significant improvements to the HOME FAST score was identified when comparing baseline (*P* = 0.78), 3-month (*P* = 0.48), and 7-month (*P* = 0.86) encounters, descriptive statistics did reveal that home modifications were made by both EG (41.0%) and CG (32.5%) participants by the 7-month encounter. It is noteworthy that while the CG did not receive any intervention visits, positive changes to the home environment were identified. The Hawthorne effect may be one explanation for this occurrence. Examples of these positive environmental changes include handrail installation, use of properly fitted footwear, improvement in lighting, and removal of floor mats and rugs.

## Discussion

This study identified that HOP-UP-PT participants had significantly reduced fall rates and improved fall risk indicators from baseline after receiving five in-person visits over 3-months followed by three monthly telerehabilitation visits with a final in-person assessment at 7-months. This is in congruence with prior reports examining outcomes of the HOP-UP-PT program and provides further support for the impact of a community-based referral source as a point of entry into the healthcare system to deliver upstream, prevention-focused care in the homes of older adults at-risk for decline [20, 21]. Additional program support comes by way of evidence suggesting that PT guided activity promotion more than doubles the likelihood that adults will achieve optimal activity dosing [49]. The individualized home-based approach is a defining feature of HOP-UP-PT and of value in reducing preventable falls given 82% of falls occur in the place where older adults reside, [9] and these individuals may be predisposed to having difficulty accessing outpatient physical therapy or community balance programming. Furthermore, when comparing those who participated in HOP-UP-PT with those who did not, differences in key fall risk variables were identified (TUG, Four Test Balance Scale, and modified SPPB).

Significant differences were most pronounced at the 3-month encounter at which the program had been delivered primarily in-person and could suggest an added positive effect of socialization with the PT during in-home visits. This suggestion is further supported by the observation that many of the risk outcomes (gait speed, 5xSTS, modified SPPB) also improved significantly at 3-months in the CG, which was an unexpected finding. Prior studies found that addressing loneliness and social isolation issues are associated with positive clinical outcomes [50, 51]. Therefore, the social component of the in-person PT visits, with or without an intervention, may have caused CG participants to make behavior changes, thereby inadvertently biasing CG results.

Falls reported by the EG (51.4%) and CG (47.2%) at baseline suggest that the SCCs were able to appropriately refer older adults at higher fall risk compared to the overall community-dwelling older adult population [50]. With a similar population and baseline fall rate to our study, Verghese et al. found that community-dwelling elders with a mean age of 80.5 years had an annual fall rate of 44% [52]. Furthermore, the HOP-UP-PT program resulted in significantly reduced falls among those in the elevated fall risk categories. This new referral model of using community-facing organizations is a novel and safe way to compliment the well-established physician-referral paradigm to access PT services. Although direct consumer access to PT care is now available throughout the US, current billing models often require physician referral and “signing off” on PTs’ plans of care. These administrative barriers may be limiting the implementation of innovative preventative services delivered by PTs or similar providers. Furthermore, to appreciate the cost savings of these prevention-focused programs, adequate and proportional payment models should be a focus for health insurance providers.

The average medical cost of a fall requiring hospitalization among US adults over the age of 60 years is $38,842 USD while those who are treated and released from the emergency department incur an average cost of $2940 USD [7]. Extrapolating the findings of this study to 100 older adults with elevated fall risk who would participate in the HOP-UP-PT program, the reduction in falls could result in a savings range of $123,500 USD to $1.6 million USD. While cost and cost savings were not the key purpose of this study, HOP-UP-PT delivery was estimated to be less than $1500 USD and suggest further examination of the downstream financial advantages that could be realized when implementing the program is warranted. Furthermore, while participants in both groups did make some positive home safety improvements, the CAPABLE program has evidence that it can reduced Medicare expenses by nearly $2800 USD per quarter [18]. Therefore, it seems feasible that synthesizing the exercise and health behavior features of the HOP-UP-PT program with an adaptive home environment modification program such as CAPABLE could bring about an even larger cost savings to older adults at highest risk for falls and future functional decline.

### Study limitations

Limitations to this study include it was not blinded to either the recipient or the provider and the randomization process was modified from standard procedures to maintain equivalent groups between SCCs. Additionally, the long-term effects on falls and fall risk was not examined in this RCT but is planned to assess the program’s enduring impact. Furthermore, variations in maneuvers measured in the Four Test Balance Scale versus the Four Stage Balance Test limited data compatibility to the SPPB and therefore the modified calculation may have resulted in less favorable outcomes. Finally, the PT data collectors individualized ‘bedside manner’, missing data points, and unanticipated participant variations common to field testing may have brought about unintended gaps and bias to the data collected.

### Future research

As this study was conducted in one Midwestern US state, future research with geographic and regional variation is warranted to increase generalizability and applicability to other populations. Additionally, inclusion of all features of the Fried Frailty Index [53] and a more robust assessment of health behaviors (e.g., sleep and water intake) would add value to future studies. Additionally, examination of fall injury severity, long-term follow up, hospitalization and institutionalization, and quality of life measures during and after HOP-UP-PT program participation is warranted. Finally, future studies examining the administrative and fiscal considerations of the program as well as investigation of the telerehabilitation and technological barriers would be of benefit.

## Conclusion

A prevention-focused multimodal program provided by PTs in older adults’ homes proved beneficial and those with the highest fall risk demonstrated a significant decrease in falls. A collaboration between PTs and community senior centers resulted in upstreaming care delivery that may reduce both the financial and personal burdens associated with falls in an older adult population. In addition to significant improvements in key fall risk outcome measures among program participants, a comparison of fall risk outcomes between participants and non-participants provided additional support for the effectiveness of this approach. The 8-fold decrease in falls observed among those at elevated fall risk who completed the HOP-UP-PT program warrants large scale implementation and research focused on healthcare cost savings.

## Supplementary Information

**Additional file 1: Supplemental Document 1.** Clinical trial protocol.

**Additional file 2: Supplemental Document 2.** Institutional Review Board informed consent.

## Data Availability

The deidentified individual participant datasets used and analyzed during the current study are available from the corresponding author on reasonable request.
