# Supplementary material for: Impact of the HOP-UP-PT program on older adults at risk to fall: a randomized controlled trial
Source: BMC Geriatr. 2021 Oct 1;21:520. doi: 10.1186/s12877-021-02450-0 (PMC8485496; doi:10.1186/s12877-021-02450-0)
Supplement: Supplementary file 1 — Additional file 1: Supplemental Document 1. Clinical trial protocol. [file 12877_2021_2450_MOESM1_ESM.pdf]

Trial Protocol

Project summary

This novel study supports the positive benefits of Home Based Older Persons Upstreaming Physical Therapy (HOP-UP-PT) to older adults identified as "at-risk" by their local senior center after participating in a prevention-focused multimodal program provided by physical therapists in their home.

General information

Protocol Title: Prevention Focused Home-Based Physical Therapy Utilizing Community Partnership Referrals
ClinicalTrials.gov Identifier: NCT04814459, 24/03/2021
Sponsor: Oakland University, 371 Wilson Boulevard, Rochester MI 48309
Principal Investigator: Sara Arena, DScPT, Oakland University, 371 Wilson Boulevard, Rochester MI 48309

Rationale & background information

The healthcare demands of the aging population will critically stress the resources of the healthcare system. There is an urgent need for preventative services for key physical indicators of the highest-risk, community dwelling older adults to avoid hospitalization and institutionalization. The program goal is to demonstrate effectiveness of preventative, in-home interventions for at-risk older adults through early identification and referral via a community center with a focus on keeping older adult's community dwelling. Prior clinical practice models of home care physical therapy have focused on home bound individuals as this has traditionally been the focus of Medicare reimbursement. This study may provide rationale for seeking prevention focused models of care in this practice setting, by utilizing tools already available and used in home care physical therapy. Furthermore, there is a gap in evidence and infrastructure for partnership between physical therapy content experts and community services employing home- and community-based preventative interventions. In the absence of such knowledge, the development of effective community/healthcare partnerships for preventative strategies will likely remain difficult.

References (of literature cited in preceding sections)

Burns ER, Stevens JA, Lee R. The direct costs of fatal and non-fatal falls among older adults - United States. J Safety Res. 2016;58:99-103.
Center for Disease Control and Prevention. Costs of Falls Among Older Adults. https://www.cdc.gov/homeandrecreationalsafety/falls/fallcost.html. Updated August 19, 2016. Accessed August 24, 2018.

Graybill EM, McMeekin P, Wildman J. Can Aging in Place Be Cost Effective? A Systematic Review. Quinn TJ, editor. PLoS One [Internet]. 2014 Jul 24;9(7):e102705. Available from: https://dx.plos.org/10.1371/journal.pone.0102705

Rowe JW, Kahn RL. Human aging: Usual and successful. Science. 1987; 237(4811):143-149.

Rowe JW, Kahn RL. Successful aging 2.0: Conceptual expansions for the 21st century. J Gerontol B Psychol Sci Soc Sci. 2015;70(4):593-596.

Florence CS, Bergen G, Atherly A, Burns E, Stevens J, Drake C. Medical Costs of Fatal and Nonfatal Falls in Older Adults. J Am Geriatr Soc [Internet]. 2018 Apr;66(4):693–8. Available from: http://doi.wiley.com/10.1111/jgs.15304

Bergen G, Stevens MR, Burns ER. Falls and Fall Injuries Among Adults Aged ≥65 Years — United States, 2014. MMWR Morb Mortal Wkly Rep [Internet]. 2016 Sep 23;65(37):993–8. Available from: http://www.cdc.gov/mmwr/volumes/65/wr/mm6537a2.htm

Ruiz S, Snyder LP, Rotondo C, Cross-Barnet C, Colligan EM, Giuriceo K. Innovative Home Visit Models Associated With Reductions In Costs, Hospitalizations, And Emergency Department Use. Health Aff [Internet]. 2017 Mar;36(3):425–32. Available from: http://www.healthaffairs.org/doi/10.1377/hlthaff.2016.1305

Study goals and objectives

The purpose of this study was to describe the outcomes of Home Based Older Persons Upstreaming Physical Therapy (HOP-UP-PT) program participants and then to compare these outcomes to non-participants. 144 participants (n=72 per group) will be randomized to either an experimental group (EG) or a control group (CG). Six Michigan senior centers will refer adults ≥ 65 years who were at-risk for functional decline or falls. Licensed physical therapists will deliver physical, environmental, and health interventions within their approved scope of practice to the EG during nine encounters (six in-person, three telerehabilitation) delivered over seven months. The CG participants are told to continue their usual physical activity routines during the same timeframe. Baseline and re-assessments are conducted at 0-, 3-, and 7-months for both the EG and CG. Descriptions and comparisons from each assessment encounter will be analyzed.

Study design

Study Type: Interventional

Actual Enrollment: 144 participants

Allocation: Randomized (Modified Block Randomization)

Intervention Model: Parallel Assignment

Experimental group (EG) participated in the 7-month HOP-UP-PT control group (CG) included only baseline, 3-month, and 7-month assessments. The CG was instructed to continue their normal level of activity throughout the 7-months after which they were offered the opportunity to receive the HOP-UP-PT program. This study reports only the 7-month time frame during which the EG and CG can be directly compared.

Masking: None (Open Label)

Primary Purpose: Prevention

Official Title: Prevention Focused Home- based Physical Therapy Utilizing Community Partnership Referrals

Actual Study Start Date: January 1, 2019

Estimated Primary Completion: Date: May 1, 2021

Estimated Study Completion Date: May 1, 2021

Eligibility Criteria

Ages Eligible for Study:	65 Years and older
Sexes Eligible for Study:	All
Gender Based:	No
Accepts Healthy Volunteers:	Yes

Inclusion Criteria:

- Greater than or equal to 65 years of age
- Senior Community Center staff identified them as 'at-risk' for decline in community dwelling status due to physical, social, economic, or community-related barriers
- Willingness to participate

Exclusion Criteria:

- Received physical therapy services within the prior two months in any setting
- Had been hospitalized within the prior two months
- Were currently receiving palliative or hospice care
- Mini Cog score less than 4 and Trail Making Part B score greater than 273 seconds
- Outcomes American College of Sports Medicine exercise pre-participation health screening indicating physician clearance needed for participation and after evaluation the physician will not clear

Arms and Interventions

Arm	Interventions/treatment
Experimental: HOP-UP-PT Program<br><br>HOP-UP-PT Program group will participate in the 7-month HOP-UP-PT program	HOP-UP-PT Program<br><br>Interventions provided to EG participants included; (1) the Otago Exercise Program (OEP) which is a well-established exercise program with evidence that it reduces falls among community-dwelling older adults, (2) motivational interviewing (MI) to optimize positive health behaviors, and (3) home and environmental modification recommendations aimed at safety. Participants were provided with and educated on the use of a wrist-worn activity tracker and an automated BP monitor unit. Finally, when follow up items were identified (e.g., orthostatic hypotension, community exercise classes), these referrals were made and documented.
No Intervention: Normal Level of Activity<br><br>Normal Level of Activity group will be instructed to continue their normal level of activity throughout the 7-months after which they will be offered the opportunity to receive the HOP-UP-PT program	

Methodology

Primary Outcome Measures:

1. Falls Report - Record of falls in prior year, and between each subsequent visit
2. Short Physical Performance Battery - The Short Physical Performance Battery (SPPB) is an assessment of the lower extremity function of an older person. It has predictive value

for hospitalization and death when an individual has lower scores (0-4) compared to higher scores (8-12). The SPPB is calculated using the collective outcomes of the Four Stage Balance Test, 5 Time Sit to Stand, and the 3-meter gait speed. Instead of using the 4 stage balance test, the 4 test balance scale was used as it is required by the Otago Exercise Program. A modified SPPB was calculated with the difference being that participants who held a semi-tandem position for 10 seconds but could not hold a tandem stance for 10 seconds only achieved a total score for that SPPB category of 2.

3. Timed Up and Go - The Timed Up and Go (TUG) is a times assessment of sit to stand transfer, 20 foot bout of ambulation with a 180 degree turn at 10 feet concluding with a stand to sit transfer.
4. STEADI Fall Risk Categorization - The STEADI Algorithm uses a combination of a screening questionnaire, review of medical history and medications, a home assessment, functional assessments, and fall frequency to stratify risk of future falls. While the STEADI Algorithm underwent revisions since the study onset, the 2017 version was utilized as a guide for key outcome metrics reported in this study.

Secondary Outcome Measures:

1. Stay Independent Questionnaire - This questionnaire scores a fall risk on a scale from zero to 14, with 4 or more indicating a possible fall risk. Zero is the lowest fall risk score, 14 is a highest fall risk score.
2. Modified Falls Efficacy Scale - Measures fear of falling for 10 indoor and 4 outdoor activities. For each statement the participant circles a number that corresponds to their level of confidence, with zero being no confidence and 10 being extreme confidence. Once all 14 items are scored, they are added for a total score maximum of 140 which is then divided by 14. A number closer to 14 equates to more confidence, and a number closer to zero equates to less confidence.
3. Health Behavior Questionnaire - Assessment of current behaviors related to physical activity, consuming fruits and vegetables, not smoking, and being at a recommended weight. Patients are categorized using the five levels of the trans-theoretical model of behavior change. The pre-contemplating stage, or not ready for change is scored a 5. The contemplation stage or considering making a change in the next 6 months is scored a four. The preparation or preparing to make the change in the next 6 months is scored a 3. The action stage indicates the participant has made the change and is scored. The maintenance stage indicates the participants have maintained the change for at least 6 months and are scored a 1.
4. Functional Comorbidity Index - Self-assessment of disease comorbidity with physical function as the outcome. 18 diagnoses are listed and if the participant has been diagnosed with the condition it would be marked at a 1 and if they do not have the condition it would be marked as a zero. Zero would be the fewest amount of co-morbidities and 18 would be the highest amount of co-morbidities.
5. HOME FAST - Assessment of participant home safety and modification needs. The tool evaluates 25 home safety domains and includes questions related to lighting, floor

surfaces, and properly fitted foot wear. Twelve of the home safety questions are scored as either Yes (indicating the recommended safety modifications was present) or No (indicating the recommended safety modifications had not been met). An additional 13 questions have an N/A option to be used in circumstances where a condition was not met (e.g., participant does not have a pet or stairs in the home). The investigators coded the responses as YES=1 point, NO= 2 points, an N/A= null with that category not being considered during data analysis. Therefore, overall HOME FAST scores that decrease over time would suggest diminished home fall and accident risks.

6. Body Mass Index - Assessment of body fat calculated from height and weight.
7. Blood Pressure - Assessment of blood pressure in supine, seated and standing for the purpose of screening for orthostatic hypotension (Measuring orthostatic blood pressure.

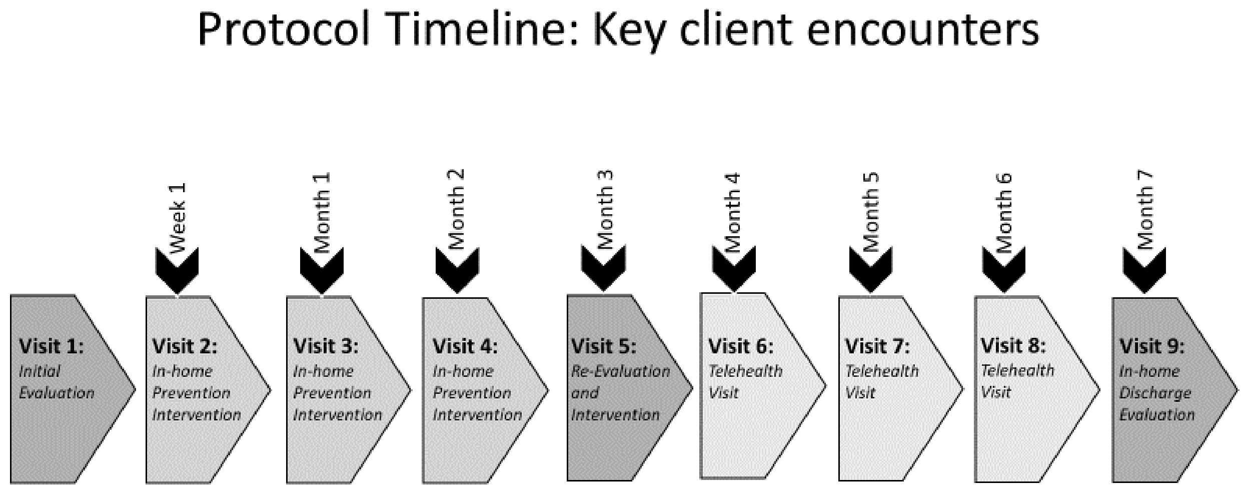

Safety considerations

Promoting activity and independence has certain risks associated with it. Although prior evidence reports very few adverse events related to the Otago Exercise Program (a component of this program), the program does require increase in physical activities such as daily walks, presenting increased opportunities for falling. Falls can have serious consequences: trauma, pain, impaired function, loss of confidence in carrying out everyday activities, loss of independence and autonomy, and even death. {Excerpted from Otago Training Manual}

Licensed physical therapists are experts in the area of safe mobility, safe exercise and fall prevention. During the evaluation and guided exercises, participants will be closely monitored at all times and the physical therapist will teach safe techniques to exercise safely. If needed, the therapist will modify exercises for safety using specialized equipment such as safety belts in accordance with physical therapy practice guidelines. If the therapist does not feel the exercise program is safe to complete, participants will not be permitted to continue the exercises as a part of this study. The physical therapist will notify the participant this occurs and what steps

should be taken, which might include calling the participants doctor or initiate a formal physical therapy regimen outside the scope of the research study.

Follow-up

The normal activity group participated in the program after the 7- months of normal phase. Additionally, a 3- and 6-month post program telephone follow up survey asked about fall occurrence and program benefits.

Data management and statistical analysis

A data management plan is in place with oversite from the Oakland University Research Office.

Descriptive statistics will be generated to analyze demographic and outcome variables. The baseline, 3- month and 7 -month encounters for the intervention and normal activity groups will be compared via a two-sample Wilcoxon test (for continuous or ordinal variables) and a Chi-squared test (for binary variables). Adjusted means will be used for interval changes, so 95% confidence intervals will be included. Statistical analysis will be performed using SAS v.9.4 (SAS Institute, Cary, NC) software for Windows with significance determined at $P < .05$. A statistician from Public Health Science, Henry Ford Health System will conduct the statistical analysis.

Quality assurance

8 hours of data collector training, monthly communication, periodic data reviews and a 3 hour data entry training class will be used to assure quality control. Additionally, the use of three non- data collectors for project oversight and a statistician outside the scope of the protocol development for data analysis added to the quality assurance of study outcomes.

Expected outcomes of the study

The investigators hypothesize older adult HOP-UP-PT participants will have reduced falls and fall risks compared to their non-participant counterparts. This will be an important contribution to the current body of evidence as it offers a novel approach that uses physical therapists and community senior center collaboration to identify at risk individuals. Furthermore, it may offer an opportunity to bridge the gap between public health and healthcare.

Dissemination of results and publication policy

Dr. Sara Arena will be the lead investigator in the efforts to dissemination study outcomes. Drs. Christopher Wilson, Lori Boright and Edward Peterson will provide critical oversight to all aspects of the dissemination. It is anticipated that the outcomes will be distributed at national physical therapy conferences and in leading peer-reviewed journal publications. Additionally,

these outcomes will help to inform the creation of a web portal that will be utilized for physical therapist program training and have community facing free resources.

Duration of the project

March 2019-May 2021

Problems anticipated

The investigators anticipate that the advanced age of the demographic planned for enrollment may have health issues outside the scope of the study that could contribute to an inability to complete all aspects of the study protocol. Additionally, it is possible some data will not be able to be ascertained as the clinical judgement of the physical therapist data collector may contribute to omission of some measures. Finally, modification to the short physical performance battery to integrate the Otago Program which uses the Four Test Balance Scale (not the Four Stage Balance Test) may result in a lower-than-actual SPPB score for all group means.

Project management

Drs. Sara Arena and Chris Wilson contributed to the study concept, design, secured study funding, data interpretation and initial drafting of the manuscript. Dr. Lori Boright contributed to data acquisition and clinical trial registry. Dr. Ed Peterson conducted data analyses. All authors contributed to the critical revisions of the manuscript for important intellectual content.

Ethics

Senior community centers will be the source of referral for this study. It is possible that underserved communities could have decreased access to senior community centers and/ or may be less willing to serve as a participant in a study.

Informed consent forms

The approved informed consent is included as Supplement 2
