# Supplementary material for: Impact of the HOP-UP-PT program on older adults at risk to fall: a randomized controlled trial
Source: BMC Geriatr. 2021 Oct 1;21:520. doi: 10.1186/s12877-021-02450-0 (PMC8485496; doi:10.1186/s12877-021-02450-0)
Supplement: Supplementary file 2 — Additional file 2: Supplemental Document 2. Institutional Review Board informed consent. [file 12877_2021_2450_MOESM2_ESM.pdf]

Consent to Participate in a Research Study

Prevention Focused Home-Based Physical Therapy Utilizing Community Partnership Referrals

Introduction

You are being asked to participate in a research study that is being done by researchers from Oakland University. This study is being done by Sara Arena, PT, DScPT, Assistant Professor and Christopher Wilson PT, DScPT, DPT, GCS, Assistant Professor. The purpose of this consent form is to let you know more about the study so you can decide whether to participate in the study or not. Please read the form carefully. You may ask questions about why the research is being done, what you will be asked to do, the possible risks and benefits, your rights as a participant, and anything else about the research or this form that is not clear. You may talk with your friends and family about this research study before making your decision. When all your questions have been answered, you can decide if you want to be in this study. This process is called 'informed consent.' If you decide to participate, you will be asked to sign this form and will receive a copy of the form.

Why is this study being done?

The purpose of this research study is to examine the effects of a prevention-based screening program and intervention strategies performed by physical therapists in your home after being referred by a community partner. Traditionally, home care physical therapist services have been initiated after a fall, injury, weakness or health issue has already occurred. This study will examine the effect of home care physical therapy preventative interventions before an injury or illness has occurred or has limited a person's ability to safely leave their home.

This study is important because as someone ages, he or she is at increased risk of having difficulty living at home due to health, medical, mobility and safety issues. For example, falls are a major public health problem because they are common in people aged 65 and older. Falls are the leading cause of injury in this age group and can have serious consequences including injury, impaired function, loss of confidence when carrying out everyday activities, loss of independence and autonomy, and even death. Approximately 33% of healthy older people will have at least one fall each year and aging increases the risk of falls and the potential for injury after a fall. Events like falls and weakness have multiple factors such as diet, medications, exercise, home environment and social support network. This study will evaluate the status of these areas in your home environment with individualized interventions to address key risk factors.

Who can participate in this study?

You are being asked to participate in the study because you have been identified through a local community center serving seniors as aged 65 or older and at risk for a decline in community-dwelling status due to physical, socioeconomic or community-related barriers. You have expressed a willingness to participate in a free home based health promotion and prevention evaluation and intervention provided by a licensed physical therapist. You would not be eligible to participate if you are currently receiving physical therapy or have received physical therapy services within the past two months at home, in a hospital, rehabilitation center, nursing home or outpatient practice setting. You would also not be eligible if you were admitted to a hospital within the prior two months. In addition, you would not be eligible to participate if you are currently receiving palliative or hospice care. Finally, you may not be eligible to participate if the evaluation by the licensed physical therapist suggests that your medical or cognitive status (as tested by an assessment called Mini-Cog and the Trail Maker part B) does not permit safe physical therapy interventions without further medical work up or physician evaluation.

Who is sponsoring this study?
This study has received grant funding from the Michigan Health Endowment Fund and the Oakland University Prevention Research Grant.

Where is this study being done?
Study procedures will be completed in your home and data analysis will occur at Oakland University.

What procedures are involved with this study?
If you agree to take part in this research study, you will be asked to do the following:

You will be visited by a physical therapist in your home at a time of your choosing. You will be randomly assigned to either start the program at the time of the initial visit, and receive 6 free in-person sessions and 5 phone calls or you will receive 2 evaluation visits and then start the full program 6 months later. Randomization will be decided using a coin flip for the first referral from each community center and alternating group assignment by household in order of consent. This will assure an equal distribution to each group.

You will receive a free comprehensive physical assessment in your home at a time of your choosing by a physical therapist licensed in the State of Michigan. The physical therapist will ask a series of questions and perform assessments of your fall risk, balance, coordination, health risk assessment, home environment and safety assessment and you will be asked about your past medical and social history as well as fill out forms to detail your health history and status. For some of these physical tests, you will be asked to walk a short distance and perform tasks that involve standing and safely challenging your balance. After this assessment and the initiation of the program, you will receive education on your results and what actions you should take to improve your health and safety at home.

In a week after your assessment, the physical therapist will educate you in an exercise regimen entitled the Otago Exercise Program. This Otago Exercise Program is a set of muscle strengthening and balance retraining exercises delivered during four or five home visits by a trained instructor who is a licensed physical therapist in the State of Michigan. You will also be instructed in use of an activity sensor called Fitbit placed on the wrist to help you track your progress with your exercise and activity. Each session after visit 2, the therapist will review your exercises, assess your health status, answer any questions or concerns you have, and collect data from the Fitbit unit. Upon your last in-person visit with the physical therapist, the previously administered health and physical function assessments will be re-administered.

In order to participate in the exercises and assessments that are a part of this study, loose fitting comfortable clothing and a pair of exercise shoes may be helpful to you. These items will not be supplied to you as a part of this study.

How long will participation in this study last?
If you are randomly assigned to start the program immediately, your overall participation in this study will last twelve months with five visits to your home over the first three months. Each in-person visit with the physical therapist is anticipated to last approximately 50-90 minutes. After your first visit with the physical therapist, you will be visited again approximately one week later and again in two to three weeks after visit two. After visit three, you will be visited approximately once a month for two months. After the five in-person visits are complete, you will be contacted via telephone by study personnel on a

monthly basis to discuss your status and progress and answer any questions that you have about your exercises and lifestyle changes. It is anticipated that the telephone discussions would be 15 minutes in duration. At the end of six months, you will be visited in the home once more for a re-evaluation, resulting in a total of six home visits. This will be followed by two additional phone calls at three and six months to discuss your status, progress and program satisfaction.

If you are randomly assigned to have a six month delay in starting the program your overall participation in this study will last eighteen months with two visits to your home over the first three months. Six months after the initial assessment you would begin the program as described above.

The researcher may stop your participation in this study at any time without your consent if the evaluation by the licensed physical therapist suggests that your medical or cognitive status does not permit safe physical therapy interventions without further medical workup or physician evaluation.

How many people will be participating in this study?
One hundred and eighty five people will be participating in this study.

What are the risks, side effects or discomforts that can be expected from participating in this study?
By taking part in this study, you may be at risk for the following:

Increasing your activity and exercise level has certain risks associated with it. Although there were very few adverse events in the research trials as a result of the Otago exercise program, it is important to be aware that the program does require an increase in movement, walking and exercise which entails increased risk for falling. Falls can have serious consequences including injuries, pain, decreased confidence or ability to perform daily activities, and even death. Licensed physical therapists are experts in the area of safe mobility, safe exercise and fall prevention. During your evaluation and guided exercises, you will be closely monitored at all times and the physical therapist will teach you safe techniques to exercise safely. If needed, the therapist will modify your exercises for safety using specialized equipment such as safety belts. If the therapist does not feel the exercise program is safe to complete, you will not be permitted to continue the exercises as a part of this study. The physical therapist will notify you if this occurs and what steps you should take, which might include calling your doctor or a formal physical therapy regimen.

A breach of confidentiality is also a possible risk. Breach of confidentiality means that it is possible that individuals not associated with this research may accidentally gain access to information that personally identifies participants. Appropriate safeguards are set in place to minimize a breach of confidentiality of your personal and medical information (e.g. researcher’s office is secure and computers and external storage devices are password protected); but no researcher can ever guarantee that this sort of breach will not occur.

Use of an automated blood pressure device requires a cuff to be placed around your upper arm, inflate with air and then deflates. Some individuals experience pressure at the site of the cuff during inflation. Although uncommon, some individuals have reported pain and bruising at the site of cuff inflation. If this situation arises, the physical therapist can measure your blood pressure with a manual device which allows for improved control in the speed and amount of cuff inflation.

With application of wearable technology that contacts the skin like the Fitbit wrist band, there is a small risk of skin irritation. If this situation arises, you would be guided to wear a sock under the ankle band to prevent direct skin contact. In addition, a Fitbit clothing clip would be offered as an alternative based on your preference.

Are there any known benefits from taking part in this study?
As someone ages, muscle strength and stability is naturally lost, but this loss does not immediately affect daily functions until a certain threshold is reached. It is possible that, when a person is near this threshold, even small gains in strength and balance can lead to preservation of daily function and safety. Investigations into the Otago Exercise Program demonstrated a 35% reduction in fall rates in home-dwelling participants older than 65. Other potential benefits of moderate physical activity are reduced death rates and improved physical and emotional health, physical function, quality of life, sleep, and sense of wellbeing.

What are the alternatives to participation in this study?
You may choose not to participate in this study.

What are the costs of taking part in the study?
There is no cost to you for participating in this study. Neither you nor your medical insurance company will be billed for any services that you receive as a part of this study.

What compensation is being provided for participation?
For taking part in this study, you will be utilizing an automated blood pressure cuff and electronic activity sensor system (called Fitbit). For your time and inconvenience you will be allowed to keep these items. If you choose to withdraw from this study at any time, you will still be allowed to keep these items. These items are valued at approximately $200.00. As this incentive is more than $75.00, your name as a research participant (without linking you to a specific research study) will be reported to the Oakland University Accounts Payable Office.

What are your rights if you participate in this study?
Your decision to participate in this study is voluntary. You may choose to leave the study at any time, or refuse to answer any questions that may be asked during the study. You will not lose any benefits to which you are otherwise entitled and your decision will not affect your present or future relationship with Oakland University, the researcher, the Oakland University Physical Therapy Department; or the local community center that referred you. If you are a student or employee at Oakland University, your decision about participation will not affect your grades or employment status.
If you would like to stop participating in this study, you should contact the researcher Sara Arena at 248-364-8682, who will provide instructions on how to withdraw from the study.
Any new information that may affect your willingness to participate in the study will be provided to you as soon as possible.

What will be done to keep my information confidential?
Every effort will be made to keep your study-related information confidential.

Personal information regarding your participation in this study may be disclosed if required by law. Also, your research records may be reviewed by the following groups:

- Regulatory authorities involved in the oversight of research (Office for Human Research Protections or other federal, state, or international regulatory agencies);
- Members or representatives of Oakland University Institutional Review Board (IRB) (in order to ensure that your rights as a research participant are being protected);

When study results are presented at professional conferences or published in professional journals, your name will not be used.

During the course of your research participation, some aspects of your health and safety may be identified that would benefit from additional follow up. In order to facilitate communication of this information, you have the opportunity to provide consent to share this information to specific parties. Possible purposes for such disclosure may include continuation of treatment or health care, referral to rehabilitation services, social service referral or community partner referral. Relevant research findings that would be disclosed includes examination results including results of medical, cognitive, or physical assessments, treatment results and recommendations, safety recommendations including needed follow up and medical follow up recommendations.

You can revoke or modify this consent at any time except to the extent that the research team has already taken action with regard to your consent (i.e. information has already been mailed or communicated). Your decision to allow information to be disclosed will not have any impact on your ability to participate in this study, only whether the results would be shared with the following parties. By initialing the box below and signing the consent form, you are providing consent for this disclosure of your research information.

_________________ Initial here to provide consent for research information to be released to the individual(s) or organizations listed below.

1. My personal physician: Name: ____
2. My referring community center
3. A designated family member or other authorized individual: Name: _________________________
4. Additional person(s) or organization(s) to whom disclosure is to be made:
    a. NAME:____
    b. RELATIONSHIP: ____
    c. PHONE (including area code): ____
    d. ADDRESS: ____
    e. FAX (including area code): ____

What do you do if you have questions about the study or the rights of research participants?
For questions about the study you may contact Sara Arena PT, DScPT at 248-364-8682 or arena@oakland.edu.

For questions regarding your rights as a participant in human subjects research, you may contact the Oakland University Institutional Review Board, 248-370-2762.

Signing the consent form
You have read (or someone has read to you) this form. You are aware that you are being asked to participate in a research study, and you understand the possible risks and potential benefits. You have had

the chance to ask questions and have had them answered to your satisfaction. You voluntarily agree to participate in this study.

You are not giving up any rights by signing this consent form. You will be given a copy of this form.

______________________________	______________________________
**Print name of participant**	**Signature of participant**
	______________________________ **AM/PM**
	**Date and time**
______________________________	______________________________
**Print name of person authorized to consent for participant**	**Signature of person authorized to consent for participant**
______________________________	______________________________ **AM/PM**
**Relationship to the participant**	**Date and time**

Investigator/Research Staff

I have explained the research to the participant or his/her representative before requesting the signature(s) above. There are no blanks in this document. A copy of this form has been given to the participant or his/her representative.

______________________________	______________________________ **AM/PM**
**Signature of person obtaining the consent**	**Date and time**

____

Print name of person obtaining the consent
